# *Salmonella* in Peripheral Lymph Nodes of Healthy Cattle at Slaughter

**DOI:** 10.3389/fmicb.2017.02214

**Published:** 2017-11-09

**Authors:** Hattie E. Webb, Dayna M. Brichta-Harhay, Mindy M. Brashears, Kendra K. Nightingale, Terrance M. Arthur, Joseph M. Bosilevac, Norasak Kalchayanand, John W. Schmidt, Rong Wang, Sophie A. Granier, Tyson R. Brown, Thomas S. Edrington, Steven D. Shackelford, Tommy L. Wheeler, Guy H. Loneragan

**Affiliations:** ^1^International Center for Food Industry Excellence, Texas Tech University, Lubbock, TX, United States; ^2^U.S. Department of Agriculture, Agricultural Research Service, U.S. Meat Animal Research Center, Clay Center, NE, United States; ^3^Laboratory for Food Safety, ANSES, Université Paris-Est, Maisons-Alfort, France; ^4^Cargill Inc., Wichita, KS, United States; ^5^Diamond V Mills, Inc., Cedar Rapids, IA, United States

**Keywords:** *Salmonella*, lymph nodes, cattle, seasonality, regionality, serotype, antimicrobial resistance phenotype

## Abstract

To more fully characterize the burden of *Salmonella enterica* in bovine peripheral lymph nodes (PLN), PLN (*n* = 5,450) were collected from healthy cattle at slaughter in 12 commercial abattoirs that slaughtered feedlot-fattened (FF) cattle exclusively (*n* = 7), cattle removed (or culled) from breeding herds (*n* = 3), or both FF and cull cattle (*n* = 2). Qualitative and quantitative methods were used to estimate prevalence and concentration of *Salmonella* in PLN. Isolates were subjected to a variety of phenotypic, serological, and molecular assays. Overall, *Salmonella* prevalence in PLN from FF and cull cattle was 7.1 and 1.8%. However, burden varied by season in that observed prevalence in PLN collected in cooler or warmer seasons was 2.4 and 8.2%, respectively. Prevalence in PLN from cull cattle in the southwest region of the US was 2.1 and 1.1% for cool and warm seasons, respectively; however, prevalence in FF PLN was far greater in that it was 6.5 and 31.1%, respectively. *Salmonella* was recovered from 289 (5.6%) PLN and 2.9% (*n* = 160) of all PLN tested had quantifiable concentrations that varied from 1.6 to 4.9 log_10_ colony forming units/PLN. The most common serotypes isolated from PLN were Montevideo (26.9%), Lille (14.9%), Cerro (13.0%), Anatum (12.8%), and Dublin (6.9%). In all, 376 unique isolates were collected from the 289 *Salmonella*-positive PLN. Antimicrobial susceptibility testing revealed the majority (80.6%) of these isolates were pansusceptible; however, 10.7% of isolates were found to be resistant to two or more antimicrobial classes. We were able to document an observed increased in prevalence of *Salmonella* in PLN during the warmer season, particularly in FF cattle from the southwest region of the US. The mechanisms underlying the observed association between season, region, and production source have yet to be elucidated. Nevertheless, these findings increase our understanding of the sources of contamination of beef products and shed light on transmission dynamics that may be useful in targeting these sources.

## Introduction

*Salmonella enterica* subspecies *enterica* (here after referred to as *Salmonella*) is an important group of foodborne pathogens resulting in an estimated 1.2 million illnesses, more than 23,000 hospitalizations, and 450 deaths in the United States (US) each year (Scallan et al., 2011). Financial losses resulting from *Salmonella* infections are considerable; the United States Department of Agriculture (USDA) Economic Research Service (ERS) reports that the total annual cost of foodborne illness attributed to *Salmonella* is $3,666,600,031 in medical expenses, loss of productivity, and cost of premature death (Economic Research Service (ERS), 2014). Common vehicles of exposure—many of which are presumably attributable to vertical transmission, fecal contamination and poor food hygiene—include eggs, raw milk and dairy products, poultry, produce, and beef (Guo et al., 2011).

Ground beef is an important vehicle for human exposure to foodborne pathogens (including *Salmonella*), and was implicated in three outbreaks of salmonellosis between 2010 and 2015 (McLaughlin et al., 2006; Centers for Disease Control and Prevention (CDC), 2012b, 2013). Authors have proposed that the carriage of *Salmonella* by cattle may contribute to the overall prevalence within ground beef products (Bosilevac et al., 2009). It is a common belief that much of the contamination of beef products results from fecal contamination of hides that in turn contaminate the carcass surface during carcass dressing. Consequently, comprehensive food safety systems have been developed and implemented into slaughter processes to mitigate risks associated with surface contamination. In laboratory settings, the same control strategies that have been implemented generally mitigate both *E. coli* O157:H7 and *Salmonella* (Wheeler et al., 2014). These interventions appear to have effectively reduced the burden of foodborne illness attributed to *E. coli* O157:H7 (Centers for Disease Control and Prevention (CDC), 2015); in 2001, the USDA Food Safety Inspection Service (FSIS) recovered *E. coli* O157:H7 from 0.9% of ground beef samples, whereas in 2014 the recovery was just 0.04%. However, the same successful outcomes have not been observed for *Salmonella* (Centers for Disease Control and Prevention (CDC), 2015). Despite the implementation of interventions, the prevalence of *Salmonella* in ground beef products has remained relatively constant and ranges between 1.6 and 4.2% depending on the size of the sample and the analysis methods employed (Bosilevac et al., 2009; Food Safety Inspection Service, 2011).

Because current in-plant intervention strategies primarily targeting surface contamination have not been successful in decreasing the burden of *Salmonella*—at least to the same extent as *E. coli* O157, alternative potential routes of contamination other than sanitation carcass dressing procedures have been investigated (Arthur et al., 2008; Haneklaus et al., 2012; Gragg et al., 2013a). Authors proposed that pathogen contamination of ground beef might also occur via incorporation of contaminated lymph nodes, particularly certain peripheral lymph nodes (PLN) (Arthur et al., 2008; Haneklaus et al., 2012; Gragg et al., 2013a; Li et al., 2015). Harborage within the PLN provides *Salmonella* protection against surface-oriented mitigation approaches, and this source of contamination would explain the greater prevalence of *Salmonella* observed in ground beef relative to beef trim destined for ground beef. In prior exploratory work, researchers found that recovery of *Salmonella* from bovine PLN is not uncommon and that likelihood of recovery might be associated with factors such as season (Gragg et al., 2013a). Given the importance of (a) *Salmonella* as a food-borne pathogen, and (b) the need to target areas of greatest burden, we set out to more fully characterize and describe the burden of *Salmonella* in PLN in healthy cattle at slaughter by season, region of the country, and production source. This information will be useful in informing risk assessors and targeted risk abatement if and where it is warranted.

## Materials and methods

**Figure 1:**
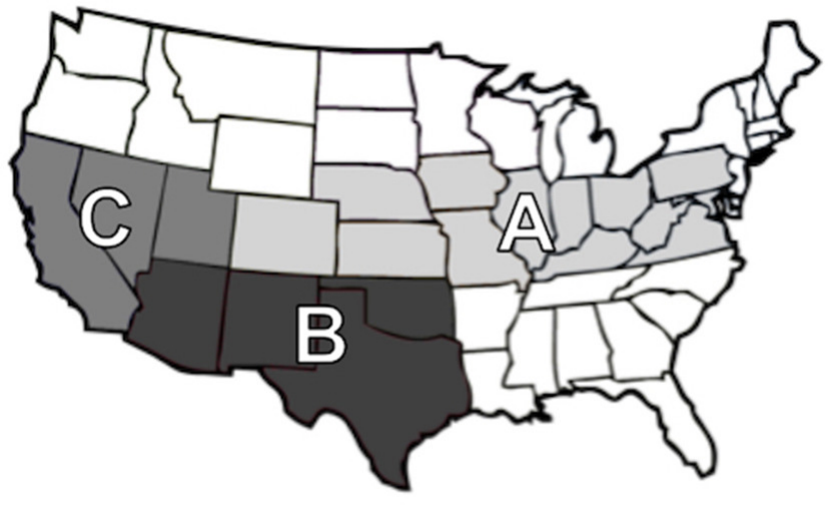
Map of regions based on geographic locations; Region A (Colorado, Illinois, Indiana, Iowa, Kansas, Kentucky, Maryland, Missouri, Nebraska, Ohio, Pennsylvania, Virginia, and West Virginia), Region B (Arizona, New Mexico, Oklahoma, and Texas), and Region C (California, Nevada, and Utah).

Subiliac PLN were collected from carcasses of cull and feedlot-fattened (FF) cattle presented for slaughter at commercial abattoirs. The subiliac was selected for it's relatively large size, the convenience with which it can be collected without affecting normal operations in commercial abattoirs, and because it has been in the PLN of choice in most prior studies. Samples were collected over approximately a 1-year period. Samples collected in spring (February through May, 2012) and winter (November and December 2012) were designated as being collected in “cooler seasons,” and those sampled in summer/fall (June through October, 2012) were designated as being collected in “warmer seasons.” The convenience sample collection included 12 commercial processing abattoirs, including: seven abattoirs that almost exclusively slaughtered cattle fattened in feedlots, three abattoirs that almost exclusively slaughtered cattle removed (or culled) from breeding herds and dairy cattle (i.e., cull cattle), and two abattoirs that slaughtered a mix of FF and cull cattle. Participating abattoirs were categorized regionally (Figure 1) as regions A, B, and C. Three times in each season, a convenience sample of 75 PLN were collected from each of the abattoirs. Following collection, PLN were shipped to either Texas Tech University or the U.S. Meat Animal Research Center for microbiological analyses. All samples were collected from carcasses of animals after post-mortem veterinary inspection at federally inspected commercial abattoirs. As such, the research described herein did not use live animals.

### Lymph node sample processing and *Salmonella* detection

Peripheral lymph nodes were processed as described previously (Brichta-Harhay et al., 2012). Briefly, after the surrounding fat and fascia were trimmed from PLN, the samples were weighed, submerged into boiling water for 3–5 s for surface sterilization, placed in a filtered sample bag (Nasco, Atlanta, GA), pulverized using a rubber mallet, and enriched in 80 mL of tryptic soy broth (TSB; Becton Dickinson, Sparks, MD). Following homogenization, samples were incubated at 25°C for 2 h and then 42°C for 12 h. Enrichments were subjected to immunomagnetic separation (IMS) using paramagnetic beads coated with antibodies to *Salmonella* (Dynabeads anti-*Salmonella*, Invitrogen, Oslo, Norway) and the IMS product was transferred to 3 mL of Rappaport-Vassiliadis (RVS; Remel, St. Louis, MO) broth, which was incubated at 42°C for 18 to 20 h. The incubated RVS broth was streaked onto xylose lysine desoxycholate (XLD; Remel, St. Louis, MO) and brilliant green sulfa (BGS; Becton Dickinson, Franklin Lakes, NJ) agar plates prior to incubation at 37°C for 18–20 h.

Quantitative analysis of *Salmonella* harborage was performed as described previously (Brichta-Harhay et al., 2012; Gragg et al., 2013a), with slight modifications as follows. For this, 1 mL of TSB-PLN homogenate was plated in duplicate to Enterobacteriaceae count plates (EB Petrifilm™; 3M Microbiology, St. Paul, MN; hereafter referred to as EB plates), prior to incubation for enrichment. Enterobacteriaceae count plates were incubated at 37°C for 22 to 26 h; following incubation, colonies were counted. For each EB plate with characteristic gas producing colonies, the plastic film cover with the thin film of agar attached, was removed from the foam backing, and then gently pressed against the surface of an XLD agar plate, essentially replica-plating the colonies present in the EB plate agar. Xylose lysine desoxycholate (XLD) plates were then incubated at 37°C for 16 h and colonies demonstrating typical *Salmonella* morphology (black colonies with a clear pink ring) were counted and up to 15 colonies per plate were selected for further confirmation. Colonies with morphology atypical of *Salmonella* on XLD were counted and the number deducted from the original count to arrive at the count used to estimate the concentration of *Salmonella* present. Peripheral lymph nodes observed to harbor *Salmonella* after the enrichment step, but at concentrations below the limit of detection of the enumeration methods (~40 colony forming units [CFU]/PLN or 1.6 log_10_ CFU), were considered to be greater than zero (i.e., *Salmonella* was present) but at a concentration <1.6 log_10_ CFU/g of PLN. In these instances a fixed value of 20 CFU/PLN—representing half of the limit of detection—was used as the concentration for data analysis. For each PLN from which *Salmonella* was recovered, the calculated log_10_ CFU/PLN was plotted vs. PLN weight (g), in order to examine contamination level as a function of PLN size (as explored by Gragg et al., 2013a), as well as to highlight seasonal and cattle production source trends.

*Salmonella* isolates were selected from enrichment plates (up to three isolates per PLN) and enumeration plates (up to 15 isolates per PLN) for serotype determination and antimicrobial susceptibility testing. Presumptive *Salmonella* isolates were confirmed using conventional polymerase chain reaction (PCR) to detect *invA* (Rahn et al., 1992; Nucera et al., 2006) and molecular serotyping methods (Herrera-Leon et al., 2004). Based on molecular results slide agglutination (O typing) and tube agglutination (flagellar H typing) methods were performed using commercial antisera (Difco, BD Diagnostic Systems, Sparks, MD) to provide a serotype. Where the aforementioned method did not provide resolution, the traditional Kaufmann-White-Le Minor scheme was used for serotyping of isolates (Grimont and Weill, 2007).

Susceptibility to 15 antimicrobial agents was determined using broth micro-dilution (Sensititre CMV2AGNF plates; TREK Diagnostic Systems, Inc., Cleveland, OH) according to manufacturer's guidelines. Isolates were categorized as susceptible or resistant to each antimicrobial based on the breakpoints established by Clinical and Laboratory Standards Institute (2013). All intermediate results were categorized as susceptible for data analysis purposes. Where breakpoints were not available (i.e., ceftiofur and azithromycin), results were interpreted using criteria recommended by Sjölund-Karlsson et al. (2011) or the National Antimicrobial Resistance Monitoring System (Food and Drug Administration (FDA), 2011). Supplementary Table 1 provides a summary of the utilized antimicrobials, abbreviations, breakpoints and antimicrobial classes.

### Statistical analysis

Exploratory data (Gragg et al., 2013a) were used to provide estimates of design prevalence for sample-size calculations. Thirty-five samples per abattoir per time point provided the ability to estimate 30% prevalence within an acceptable margin of error with 95% confidence. Further, 70 samples per plant per season was sufficient to detect *Salmonella* in one or more PLN with greater than 95% confidence, given a low-end design prevalence of 5%. Assuming β = 0.2 and α = 0.05, the sample size utilized was sufficient to detect relative differences of 50% or greater between seasons, regions, and production sources given a design prevalence of 30% (i.e., in Texas during the summer/fall season) and 5% while allowing for within-abattoir clustering.

**Figure 2:**
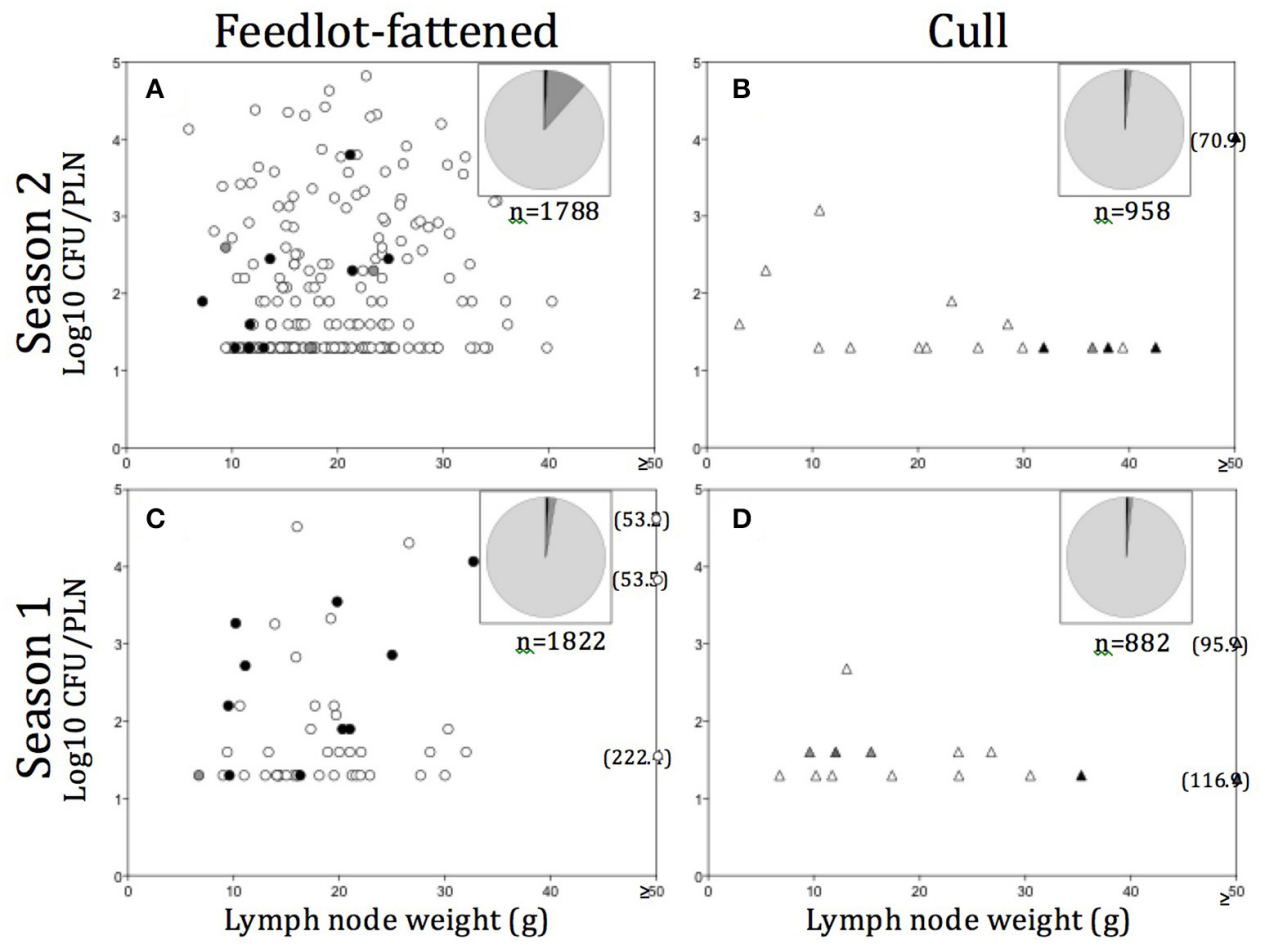
**(A–D)** Distribution of *Salmonella* contaminated peripheral lymph nodes (PLN) plotted as *Salmonella* contamination level (log_10_ CFU/PLN) vs. PLN weight in grams (g), by season and production source. Numbers in parentheses indicate PLN weight (g) for those found ≥50 g. Pie charts in each **(A–D)** represent the total number of PLN tested in each season/production source, with light gray representing the proportion tested but negative, medium gray the proportion found positive but not enumerable for *Salmonella*, and dark gray, the proportion enumerable (>1.6 log10 CFU/PLN). Filled in symbols (O, Δ) indicate particular serotypes observed and include: black, *S*. Dublin; medium gray, *S*. Newport; light gray, *S*. Typhimurium; empty, other serotypes.

A variety of models were constructed to evaluate the association of *Salmonella* prevalence with season, region, and production source. Generalized linear models were constructed assuming an over dispersed Poisson distribution; an over dispersion parameter was forced into the models to inflate the variance associated with the point estimates. For each sample set, n (the number of PLN from which *Salmonella* was recovered) was the response variable and the total number of PLN in the sample size (*n* = ~75) was log-transformed and used as the offset variable. In situations where PLN were harvested from both cull and FF animals in the plant during the same sample collection day, the samples were separated into sets based on the reported production source from which the PLN was harvested during slaughter. Fixed main effects were season, region, and production source. Because of model instability, it was not possible to evaluate the three-way interaction of the main effects. Consequently, and after visual evaluation of the crude means, a main effect of production source was tested and the two way interactions between season and region were evaluated in separate models for cull and FF cattle. Crude means and model estimates were summarized and presented in tabular formats. SAS software (version 9.4, SAS Institute Inc., Cary, NC, USA) was used to perform the statistical analyses. Enumeration data were plotted as total estimated log_10_ CFU/PLN vs. PLN weight in grams (Figure 2). Data plots were constructed using Prism 5.0d, GraphPad Software, Inc. (www.graphpad.com, San Diego, CA).

## Results

### *Salmonella* prevalence and enumeration

A total of 5,450 subiliac PLN were collected from cull and FF cattle presented for harvest in three regions of the US, across 11 months. Table 1 provides a summary of the number of sample sets, total number of PLN tested by season, region, and production source the number of *Salmonella* positive PLN, as well as the crude *Salmonella* prevalence of PLN collected by season, region, and production source. The overall crude prevalence of *Salmonella* in PLN from cull and FF animals was 1.8% (33 of 1,840) and 7.1% (256 of 3,610), respectively. The overall crude prevalence of *Salmonella* in PLN from the cooler season and warmer season were 2.4% (64 of 2,704) and 8.2% (225 of 2,746), respectively. *Salmonella* prevalence in PLN from cull animals was generally low in every region throughout 11-months (cooler season, 1.7%; warmer season, 1.9%) while that in FF cattle PLN was found to be low in the cooler season (2.7%), yet peaked during the warmer season (11.6%). Abattoirs sampled from Region B that slaughtered cull animals demonstrated a similar prevalence of *Salmonella* during both seasons (cooler season, 2.1% [adjusted 95% confidence interval 0.64–7.20%]; warmer season, 1.1% [adjusted 95% confidence interval 0.19–5.88%]). PLN collected from FF cattle originating from Region B abattoirs had a prevalence of *Salmonella* in the cooler and warmer seasons of 6.5% (adjusted 95% confidence interval 3.24–13.16%) and 31.1% (adjusted 95% confidence interval 22.64–42.59%), respectively.

**Table 1:** *Salmonella* percent prevalence in subiliac peripheral lymph nodes (PLN) of feedlot-fattened (FF) and cull cattle at harvest by region and season.

**Season**	**Cull cattle PLN**			**All Cull**	**FF cattle PLN**			**All FF**	**Overall by season**
	**Region A**	**Region B**	**Region C**		**Region A**	**Region B**	**Region C**		
**COOLER SEASON**									
Sample sets	1	9	3	13	12	9	5	26	39
Number of PLNs	76	561	245	882	892	551	379	1,822	2,704
Positive (*n* =)	0	12	3	15	2	36	11	49	64
Mean %	0	2.14	1.22	1.70	0.22	6.53	2.90	2.69	2.37
**WARMER SEASON**									
Sample sets	2	9	3	14	10	9	6	25	39
Number of PLNs	145	567	246	958	754	570	464	1,788	2,746
Positive (*n* =)	7	6	5	18	8	177	22	207	225
Mean %	4.83	1.06	2.03	1.88	1.06	31.05	4.74	11.52	8.19
**OVERALL BY REGION**									
Sample sets	3	18	6	27	22	18	11	51	78
Number of PLNs	221	1,128	491	1,840	1,646	1,121	843	3,610	5,450
Positive (*n* =)	7	18	8	33	10	213	33	256	289
Mean %	3.17	1.60	1.63	1.79	0.61	19.0	3.91	7.09	5.3

*Collection seasons were defined as cooler weather months (February–May and November–December, 2012) and the warmer weather months (June–October, 2012). Regions are based on geographic locations including: Region A (Colorado, Illinois, Indiana, Iowa, Kansas, Kentucky, Maryland, Missouri, Nebraska, Ohio, Pennsylvania, Virginia, and West Virginia), Region B (Arizona, New Mexico, Oklahoma, and Texas) and Region C (California, Nevada, and Utah)*.

In all, *Salmonella* was recovered from 289 (5.3%) of the 5,450 PLN collected. Of the PLN from which we recovered *Salmonella*, we were able to quantify the *Salmonella* concentration in 55.4% (*n* = 160, or 2.9% of all PLN tested). As demonstrated in Figures 2A–D, the enumerable concentrations ranged from 1.6 (the limit of quantification) to 4.9 log_10_ CFU/PLN, with 17.6% (*n* = 51) of positive PLN harboring *Salmonella* at concentrations >3.0 log_10_ CFU/PLN. Closer examination of the distribution of PLN harboring higher concentrations of *Salmonella* revealed that FF cattle in the warmer season (Season 2) were the major contributor to this category, representing 76.5% of enumerable PLN containing >3.0 log_10_ CFU/PLN. These PLN were predominantly found contaminated with pansuseptible *S*. Montevideo and *S*. Anatum and were isolated from FF cattle at harvest in Region B (Figure 2C). Two additional noteworthy observations were: (1) PLN harboring serotypes Typhimurium or Newport were predominantly from cull cattle (Table 2), and that *Salmonella* concentrations in these PLN were generally low (i.e., ≤1.6 log_10_ CFU/PLN; Figures 2B,D); and (2) PLN contaminated with *S*. Dublin were frequently at concentrations >2.0 log_10_ CFU/PLN and were predominantly contributed by FF cattle at harvest in Region C (Figures 2A,C and Table 2).

**Table 2:** Prevalence of *Salmonella* serotypes isolated from subiliac peripheral lymph nodes (PLN) of cull and feedlot-fattened (FF) cattle at harvest.

**Serotype**	**FSIS ground beef prevalence^a^**	**Region A**		**Region B**		**Region C**		**Total by serotype**
		**Cull**	**FF**	**Cull**	**FF**	**Cull**	**FF**	
Montevideo	1	–	–	2.8	27.9	–	1.8	26.9
Lille		–	–	2.8	15.9	–	0.3	14.9
Cerro	8	13.9	–	–	12.9	–	−	13.0
Anatum	3	–	0.9	16.7	10.0	–	1.5	12.8
Dublin	11	–	–	–	0.9	13.9	5.3	6.9
Kentucky	7	–	–	–	4.4	–	–	4.0
Mbandaka	6	–	–	8.4	3.2	–	0.3	4.0
Muenster	5	–	1.5	2.8	1.8	–	–	3.1
Meleagridis	13	–	0.3	–	1.2	–	1.8	2.9
Typhimurium	2	–	0.3	5.6	0.6	5.6	–	1.9
Brandenberg		–	–	5.6	1.2	−	−	1.6
Lubbock		–	–	–	1.8	–	–	1.6
Nontypable		–	–	2.8	0.6	–	0.6	1.3
Litchfield		–	–	2.8	0.6	–	–	0.8
Livingstone		–	–	–	0.9	–	–	0.8
Derby		–	–	5.6	–	–	–	0.5
Elmorane		–	0.6	–	–	–	–	0.5
London		5.6	–	–	–	–	–	0.5
Muenchen		–	–	–	0.6	–	–	0.5
Newport	4	–	–	2.8	–	2.8	–	0.5
Agona	9	–	–	−	0.3	–	–	0.3
Cubana		–	–	–	–	–	0.3	0.3
O4;I;-		–	–	–	0.3	–	–	0.3
Total percent by region and production source		19.4	3.5	58.3	84.7	22.2	11.7	

*Isolates collected from n = 289 positive PLN (n = 376 total isolates; cull n = 36 isolates from 33 PLN; FF n = 340 isolates from 256 PLN). Percentages calculated as the number of isolates of each serotype observed in each region, divided by the total number of isolates from the production source (n = 36 for cull and n = 340 for FF)*.

a *Ranking of Salmonella serotypes isolated from ground beef as determined by FSIS testing from 1998 to 2006 (average of 22,554 samples tested and 2.87% positive each year)*.

### *Salmonella* serotypes and antimicrobial susceptibility phenotypes

This study resulted in 376 unique *Salmonella* isolates from 289 PLN found positive for *Salmonella*. Isolation of multiple unique *Salmonella* serotypes from a single PLN occurred with 56 samples—4 of cull animal origin and 52 of FF animal origin, with most of the latter originating from the same abattoir in Region B. Occasions where 2, 3, or 4 *Salmonella* serotypes were isolated from an individual PLN occurred 45, 10, and 1 time(s) respectively. In all 22 serotypes were identified (Table 2); the majority (74.5%) of which were serotypes Montevideo (26.9%), Lille (14.9%), Cerro (13%), Anatum (12.8%), and Dublin (6.9%).

Antimicrobial susceptibility phenotyping identified most isolates, 80.6% (303 of 376) as susceptible to all antimicrobial agents tested. As summarized in Table 3, isolates resistant to two or more antimicrobial classes were infrequently observed (10.7%, *n* = 40). Tetracycline, sulfisoxazole, streptomycin, and chloramphenicol represented the most common antimicrobials to which *Salmonella* demonstrated resistance. *Salmonella* isolates were less frequently resistant to gentamicin, ciprofloxacin, nalidixic acid, and sulfamethoxazole-trimethoprim. Of the 22 serotypes observed in this study, Dublin was the serotype most frequently found resistant to one or more antimicrobials (24 of 26 isolates) and had the greatest diversity of resistance phenotypes (Table 3). Conversely, *Salmonella* serotypes Cerro, Lille, Anatum, and Montevideo predominantly demonstrated limited resistance phenotypes and were generally pan-susceptible.

**Table 3:** Percent of antimicrobial resistance phenotypes among the *Salmonella* serotypes isolated from subiliac peripheral lymph nodes (PLN) of cull and feedlot-fattened (FF) cattle at harvest.

**No. of classes**	**Resistance Phenotype**	**Montevideo**	**Typhimurium**	**Anatum**	**Newport**	**Muenster**	**Cerro**	**Dublin**	**Lille**	**Other**
6	AMP AUG AXO CHL CIP FOX NAL FIS STR TET TIO	0.3	–	–	–	–	–	1.4	–	–
5	AMP AUG AXO CHL FOX KAN FIS STR TET TIO	–	–	–	–	–	–	1.6	–	–
5	AMP AUG AXO CHL FOX SXT FIS STR TET TIO	–	–	–	–	–	–	0.3	–	–
5	AMP AUG AXO CHL FOX KAN FIS STR TET	–	–	–	–	–	–	0.3	–	–
5	AMP AUG AXO CHL FOX FIS STR TET TIO	–	0.8	–	0.3	–	–	1.3	–	–
5	AMP AUG AXO CHL KAN FIS STR TET	–	–	–	–	–	–	0.3	–	–
5	AZI CHL SXT KAN FIS STR TET	–	–	0.3	–	–	–	0.3	–	–
5	CHL KAN CIP NAL FIS STR TET	–	–	–	–	–	–	0.3	–	–
5	CHL CIP NAL FIS STR TET	–	–	–	–	–	–	0.3	–	–
4	CHL FIS STR TET	–	–	–	–	–	–	0.5	–	–
3	FIS STR TET	–	–	–	–	1.1	–	–	–	–
3	CHL FIS TET	–	–	–	–	–	–	–	–	0.3
2	AZI CIP NAL	0.3	–	–	–	–	–	–	–	–
2	STR TET	0.3	–	–	–	–	–	–	–	–
2	AZI TET	–	–	–	–	–	–	–	–	0.5
1	FIS	–	–	–	–	–	–	–	–	0.3
1	AZI	3.2	–	–	–	–	–	–	–	–
1	TET	2.4	–	0.3	–	–	0.3	–	0.5	1.6
1	AMP AUG AXO FOX TIO	–	0.3	–	–	–	–	–	–	–
0	Pansusceptible	20.5	0.8	12.2	0.3	2.2	12.8	0.5	14.4	17.0
Percent from cull-cattle PLN (*n* = 36)		0.3	1.1	1.6	0.5	0.3	1.3	1.3	0.3	2.9
Percent from FF-cattle PLN (*n* = 340)		26.6	0.8	11.2	0	2.9	11.7	5.6	14.6	17.0
Total number of isolates (*n* = 376)		101	7	48	2	12	49	26	56	75

*AMP, ampicillin; AUG, augmentin; AXO, ceftriaxone; AZI, azithromycin; CHL, chloramphenicol; SXT, trimethoprim-sulfamethoxazole; KAN, kanamycin; CIP, ciprofloxacin; FOX, cefoxitin; NAL, nalidixic acid; FIS, sulfisoxazole; STR, streptomycin; TET, tetracycline; TIO, ceftiofur; GEN, gentamicin*.

## Discussion

Previous exploratory studies established that cattle PLN can harbor *Salmonella* (Arthur et al., 2008; Haneklaus et al., 2012; Gragg et al., 2013a). As such, *Salmonella* may have the potential to circumvent in-plant carcass surface interventions and ultimately represents a human public health burden through PLN inclusion in ground beef product (Koohmaraie et al., 2012; Li et al., 2015). In the study reported here, we were able to more fully characterize and describe the burden across multiple variables. In particular, it is clear that the highest burden (and possible risk) associated with *Salmonella* harborage in PLN appears to be limited to particular seasons of the year, regions of the country, and cattle production sources. These data show the prevalence of *Salmonella* in PLN from FF cattle from Region B during the warmer season (31.1%) is responsible for driving the prevalence statistics for FF cattle higher. In fact, FF cattle PLN sampled in Regions A (1.1%) and C (4.8%) remained relatively low with values similar to those observed for cull cattle PLN. Moreover, examination of the serotypes isolated from PLN of FF cattle, especially from Region B, in comparison with serotypes most commonly isolated from ground beef (Table 2), illustrates the degree to which bovine PLN are likely a source of ground beef contaminated by *Salmonella*. With *Salmonella* concentrations ranging from 3.0 to 4.9 log_10_ CFU/PLN in 17.6% of positive PLN in this study—and the possibility of *Salmonella* harborage in multiple PLN in a given carcass (Gragg et al., 2013b)—the potential for this source of *Salmonella* to contaminate ground beef is substantial (Li et al., 2015).

An important consideration in the assessment of risk for human illness with regard to *Salmonella* infection is serotype and antimicrobial resistance. Some *Salmonella* serotypes are more likely than others to cause severe infection in humans (Jones et al., 2008; Suez et al., 2013). A diverse set of serotypes was isolated in this study; however, the dominant serotypes identified are either infrequently implicated in laboratory-confirmed cases of human salmonellosis (as in the case of Anatum, Lille, and Cerro), or when they are (as in the case of Montevideo) the sources identified are often produce, spices, cheese, or poultry products, as opposed to beef. Despite their documented presence in ground beef, serotypes such as Montevideo and Anatum may pose less of a risk to human health when present in this commodity than serotypes Typhimurium and Newport. *Salmonella* serotypes Typhimurium and Newport are observed less frequently in bovine PLN surveys, as reported here and in previous studies (Gragg et al., 2013a,b). However, the increased risk of the presence of these serotypes in ground beef resulting in human illness is evidenced by a number of ground beef related outbreaks attributed to serotypes Typhimurium and Newport (Schneider et al., 2011; Centers for Disease Control and Prevention (CDC), 2012b, 2013). In addition, FoodNet data implicate *S*. Typhimurium and *S*. Newport as two of the three serotypes responsible for the majority of laboratory confirmed human salmonellosis cases across 10 sites in the US (Centers for Disease Control and Prevention (CDC), 2012a).

When human salmonellosis does occur, it is often self-resolving and only requires care to prevent dehydration. Nevertheless, in approximately 5% of cases—mostly in immune-compromised individuals—life threatening extra-gastrointestinal infections by *Salmonella* may occur and antimicrobial treatment is then required (Acheson and Hohmann, 2001). Recommended treatment options for salmonellosis include beta-lactams (i.e., penicillin or third generation cephalosporins) or fluoroquinolones (not recommended for children)—and alternative treatment recommendations include sulfamethoxazole-trimethoprim or azithromycin. A majority of our unique strains were pansusceptible; however, 14.3% were resistant to at least one of the antimicrobial classes recommended for treatment of life threatening salmonellosis. Also worth noting, 1.7% of the isolates were resistant to both beta-lactams and fluoroquinolones—which is certainly of concern.

In keeping with this line of thought, the observation here that *S*. Dublin was detected at enumerable concentrations in PLN of both FF and cull cattle at harvest in Region C (Figure 2 and Table 2)—and was often multi-drug resistant—was unexpected and warrants further investigation. *Salmonella* Dublin is a cattle-adapted serotype that is commonly detected in ground beef testing programs (Doerscher et al., 2015; Food Safety and Inspection Service (FSIS), 2015). A recent report brought to light the incidence rate of reported *S*. Dublin infections in humans has been steadily rising in the US since the 1960s, and that these are often cases of bloodstream infections and hospitalization (Harvey et al., 2017). These observations highlight the need for an increased understanding of genes that contribute to *Salmonella's* success as a pathogen in the human host.

Caution should be taken in interpretation of seasonal-, regional-, and production source-differences observed in the data presented herein, particularly with respect to inferring causal relationships. It is possible that a seasonal association might have been a causal relationship. Alternatively, the association might have been confounded by an unmeasured variable, such as an atypical regional weather event in, for example, Region B. Production source also reflects many unmeasured variables—for example, age of animal at slaughter; in the US, a vast majority of FF cattle are slaughtered at less than 2 years of age, whereas animals culled from breeding herds are typically older with a much wider variation in age. Further complicating the inference on causal relationships is that, in general, animals can only be sampled once, as PLN collection occurs post mortem except in controlled experimental situations. Despite these limitations, consideration of data reported elsewhere infers there are indeed likely causal associations with season, region, and production sources. Gragg et al. (2013a) reported similar associations of *Salmonella* burden in PLN to those observed here, and *Salmonella* was recovered from a substantial proportion of PLN collected from FF cattle presented for slaughter in Texas or Veracruz, Mexico (Sofos et al., 1999; Haneklaus et al., 2012; Gragg et al., 2013b; Brown et al., 2015; Cernicchiaro et al., 2016). Moreover, *Salmonella* is routinely recovered from the feces of cattle in the southern portion of the US (Kunze et al., 2008; Rodriguez-Rivera et al., 2016), yet remarkably, recovery of *Salmonella* from cattle feces decreases to the north (Wells et al., 2001; Sorensen et al., 2002; Rao et al., 2010; Morley et al., 2011). While these other reports do not preclude confounding of season, region, or production source in the data reported herein, taken together, they do add support that there are indeed predictable differences in *Salmonella* burden across regions and seasons, at least within North America.

The *Salmonella* distribution reported herein can aid in identifying production sources that are more likely contributors of *Salmonella* that may be more important to human health. Targeted removal of large PLN (i.e., the subiliac, superficial cervical, and popliteal) from cattle at harvest is a possible candidate for control; however, removing PLN in the slaughter process is a difficult and imprecise task. Given the limitations of PLN removal in the dressing process, as well as the sporadic nature of PLN harborage, the need for a more precise and efficient mitigation scheme is evident, and yet such methods remain to be defined. Post-harvest strategies, such as irradiation, could decrease *Salmonella* prevalence in ground beef products, though many consumers have not yet accepted the concept of using methods such as these. Therefore, pre-harvest interventions may be a more practical. Notably, preliminary research testing of a commercially available *Salmonella* vaccine suggests this form of intervention may decrease the duration of infection in PLN for specific serotypes, in this case Newport (Edrington et al., 2013b). Further research is needed to evaluate the efficacy of these interventions for decreasing the incidence or duration of infection in bovine PLN.

Ultimately, the key to understanding the variation observed in *Salmonella* harborage in bovine PLN may lay in defining the implications of production source differences in pre-harvest practices. It is unclear by which route *Salmonella* enters the animal and then is captured within the PLN. One possible explanation is that it escapes the gastrointestinal tract and mesenteric lymph nodes, and then disseminates systemically. Hanson et al. (2016) reported evidence for vertical transmission from the dam to her fetus. Further, serotypes recovered from PLN can vary within an animal, but more closely resembles serotypes recovered from the hides of animals, whereas serotypes recovered from mesenteric lymph nodes more closely resemble those recovered from the feces (Gragg et al., 2013b). It is conceivable, therefore, that a transdermal route of infection could result in accumulation of *Salmonella* within PLN that receive lymph from the integument. Abrasions or external parasites such as biting insects might, therefore, contribute to the observed burden of Salmonella in PLN (Edrington et al., 2013a,b; Olafson et al., 2016). It is also possible that all of these proposed routes of infection contribute to the burden of *Salmonella* in PLN; the data presented here illustrate the differences observed in serotype, prevalence, and concentration among FF and cull cattle; these data make it tempting to suggest that harborage of *Salmonella* in PLN of cull cattle may be a “remnant” of a systemic infection from which the animal recovered—as evidenced by low *Salmonella* concentrations in PLN with serotypes Typhimurium and Newport. Further, that observed with FF cattle may be more so the result of recent transdermal infection via abrasions or external parasites. Factors such as management (i.e., pest control, animal health best practices, etc.), age of animal at slaughter, and nutrition are all possible drivers of the illustrated differences. Knowledge gaps in the implications of pre-harvest practices ought to be addressed, as they will be instrumental in developing effective and practical solutions to mitigate the food safety risks associated with harborage of *Salmonella* in bovine lymph nodes.

## Author contributions

Conceived and designed the experiments: GL, TE, and DB-H. Performed the experiments: HW, TB, DB-H, TA, JB, NK, JS, RW, SS, and TW. Analyzed the data and wrote the paper: HW, DB-H, and GL. Critical revision of the paper for important intellectual content: all authors.

### Conflict of interest statement

The authors declare that the research was conducted in the absence of any commercial or financial relationships that could be construed as a potential conflict of interest.

## References

[B1] Acheson D. Hohmann E. L. (2001). Nontyphoidal salmonellosis. Clin. Infect. Dis. 32, 263–269. 10.1086/318457 11170916

[B2] Arthur T. M. Brichta-Harhay D. M. Bosilevac J. M. Guerini M. N. Kalchayanand N. Wells J. E.. (2008). Prevalence and characterization of *Salmonella* in bovine lymph nodes potentially destined for use in ground beef. J. Food Protect. 71, 1685–1688. 10.4315/0362-028X-71.8.1685 18724765

[B3] Bosilevac J. M. Guerini M. N. Kalchayanand N. Koohmaraie M. (2009). Prevalence and characterization of Salmonellae in commercial ground beef in the United States. Appl. Environ. Microbiol. 75, 1892–1900. 10.1128/AEM.02530-08 19201965 PMC2663200

[B4] Brichta-Harhay D. M. Arthur T. M. Bosilevac J. M. Kalchayanand N. Schmidt J. W. Wang R.. (2012). Microbiological analysis of bovine lymph nodes for the detection of *Salmonella enterica*. J. Food Protect. 75, 854–858. 10.4315/0362-028X.JFP-11-434 22564933

[B5] Brown T. Edrington T. Loneragan G. Hanson D. Malin K. Ison J.. (2015). Investigation into possible differences in Salmonella prevalence in the peripheral lymph nodes of cattle derived from distinct production systems and of different breed types. J. Food Protect. 78, 2081–2084. 10.4315/0362-028X.JFP-15-198 26555532

[B6] Centers for Disease Control and Prevention (CDC) (2013). Multistate Outbreak of Salmonella Typhimurium Infections Linked to Ground Beef [Online]. Available online at: http://www.cdc.gov/salmonella/typhimurium-01-13/ (Accessed August 28 2013).

[B7] Centers for Disease Control and Prevention (CDC) (2012a). Foodborne Diseases Active Surveillance Network (FoodNet), Table 5 FoodNet–Number and INCIDENCE of Salmonella Infections by Serotype 2011 [Online]. Available online at: http://www.cdc.gov/foodnet/data/trends/tables/table~5.html (Accessed April 11 2013).

[B8] Centers for Disease Control and Prevention (CDC) (2012b). Multistate Outbreak of Human Salmonella Typhimurium Infections Linked to Ground Beef [Online]. Available online at: http://www.cdc.gov/salmonella/typhimurium-groundbeef/020112/index.html (Accessed August 28 2013).

[B9] Centers for Disease Control and Prevention (CDC) (2015). Preliminary incidence and trends of infection with pathogens transmitted commonly through food—foodborne diseases active surveillance network, 10 U.S. Sites, 2006–2014. Morb. Mortal. Week. Report 64, 495–499. PMC4584825 25974634

[B10] Cernicchiaro N. Ives S. E. Edrington T. S. Nagaraja T. G. Renter D. G. (2016). Efficacy of a *Salmonella* Siderophore receptor protein vaccine on fecal shedding and lymph node carriage of *Salmonella* in commercial feedlot cattle. Foodb. Pathogens Dis. 13, 517–525. 10.1089/fpd.2016.2129 27304488

[B11] Clinical and Laboratory Standards Institute (2013). Performance Standards for Antimicrobial Susceptibility Testing; Twenty-Third Informational Supplement. Wayne, PA: Clinical and Laboratory Standards Institute.

[B12] Doerscher D. R. Lutz T. L. Whisenant S. J. Smith K. R. Morris C. A. Schroeder C. M. (2015). Microbiological testing results of boneless and ground beef purchased for the national school lunch program, 2011 to 2014. J. Food Protect. 78, 1656–1663. 10.4315/0362-028X.JFP-15-101 26319719

[B13] Economic Research Service (ERS) (2014). Cost Estimates of Foodborne Illnesses [Online]. Available online at: http://www.ers.usda.gov/data-products/cost-estimates-of-foodborne-illnesses.aspx (Accessed February 1 2016)

[B14] Edrington T. S. Loneragan G. H. Genovese K. J. He H. Callaway T. R. Anderson R. C.. (2013a). Development of a transdermal *Salmonella* challenge model in calves. J. Food Protect. 76, 1255–1258. 10.4315/0362-028X.JFP-12-317 23834802

[B15] Edrington T. S. Loneragan G. H. Hill J. Genovese K. J. Brichta-Harhay D. M. Farrow R. L.. (2013b). Development of challenge models to evaulate the efficacy of a vaccine to reduce carriage of *Salmonella* in peripheral lymph nodes of cattle. J. Food Protect. 76, 1259–1263. 10.4315/0362-028X.JFP-12-319 23834803

[B16] Food and Drug Administration (FDA) (2011). National Antimicrobial Resistance Monitoring System- Enteric Bacteria (NARMS): 2012 Retail Meat Report. Rockville, MD: U.S. Department of Health and Human Services, FDA.

[B17] Food Safety and Inspection Service (FSIS) (2015). Serotypes Profile of Salmonella Isolates from Meat and Poultry Products: January 1998 through December 2013 [Online]. Available online at: http://www.fsis.usda.gov/wps/wcm/connect/c7b5903c-8e8b-4f85-9b5c-12eaf990d2dd/Salmonella-Serotype-Annual-2013.pdf?MOD=AJPERES (Accessed March 14 2016).

[B18] Food Safety and Inspection Service (2011). NARMS Retail Meat Report, 2011. Washington, DC: U.S. Food and Drug Administration

[B19] Gragg S. E. Loneragan G. H. Brashears M. M. Arthur T. M. Bosilevac J. M. Kalchayanand N.. (2013a). Cross-sectional study examining *Salmonella enterica* carriage in subiliac lymph nodes of cull and feedlot cattle at harvest. Foodb. Pathogens Dis. 10, 368–374. 10.1089/fpd.2012.1275 23566273 PMC3696922

[B20] Gragg S. E. Loneragan G. H. Nightingale K. K. Brichta-Harhay D. M. Ruiz H. Elder J. R.. (2013b). Substantial within-animal diversity of *Salmonella* isolates from lymph nodes, feces, and hides of cattle at slaughter. Appl. Environ. Microbiol. 79, 4744–4750. 10.1128/AEM.01020-13 23793628 PMC3719521

[B21] Grimont P. A. D. Weill F.-X. (2007). Antigenic Formulae of the Salmonella Serovars, 9th Edn.. Paris: WHO Collaborating Center for Reference and Research on Salmonella, Institut Pasteur Available online at: http://www.pasteur.fr/sante/clre/cadrecnr/salmoms/WKLM_En.pdf

[B22] Guo C. F. Hoekstra R. M. Schroeder C. M. Pires S. M. Ong K. L. Hartnett E.. (2011). Application of bayesian techniques to model the burden of human salmonellosis attributable to U.S. food commodities at the point of processing: adaptation of a Danish model. Foodb. Pathog. Dis. 8, 509–516. 10.1089/fpd.2010.0714 21235394 PMC3123837

[B23] Haneklaus A. N. Harris K. B. Griffin D. B. Edrington T. S. Lucia L. M. Savell J. W. (2012). *Salmonella* prevalence in bovine lymph nodes differs among feedyards. J. Food Protect. 75, 1131–1133. 10.4315/0362-028X.JFP-11-530 22691483

[B24] Hanson D. Loneragan G. Brown T. Nisbet D. Hume M. Edrington T. (2016). Evidence supporting vertical transmission of *Salmonella* in dairy cattle. Epidemiol. Infect. 144, 962–967. 10.1017/S0950268815002241 26419321 PMC4825103

[B25] Harvey R. R. Friedman C. R. Crim S. M. Judd M. Barrett K. A. Tolar B.. (2017). Epidemiology of *Salmonella enterica* Serotype Dublin Infections among Humans, United States, 1968–2013. Emerg. Infect. Dis. 23:1493. 10.3201/eid2309.170136 28820133 PMC5572876

[B26] Herrera-Leon S. McQuiston J. R. Usera M. A. Fields P. I. Garaizar J. Echeita M. A. (2004). Multiplex PCR for distinguishing the most common phase-1 flagellar antigens of *Salmonella* spp. J. Clin. Microbiol. 42, 2581–2586. 10.1128/JCM.42.6.2581-2586.2004 15184437 PMC427890

[B27] Jones T. F. Ingram L. A. Cieslak P. R. Vugia D. J. Tobin-D'Angelo M. Hurd S.. (2008). Salmonellosis outcomes differ substantially by serotype. J. Infect. Dis. 198, 109–114. 10.1086/588823 18462137

[B28] Koohmaraie M. Scanga J. A. De la Zerda M. J. Koohmaraie B. Tapay L. Beskhlebnaya V.. (2012). Tracking the sources of *Salmonella* in ground beef produced from Nonfed Cattle. J. Food Protect. 75, 1464–1468. 10.4315/0362-028X.JFP-11-540 22856570

[B29] Kunze D. J. Loneragan G. H. Platt T. M. Miller M. F. Besser T. E. Koohmaraie M.. (2008). *Salmonella enterica* burden in harvest-ready cattle populations from the southern high plains of the United States. Appl. Environ. Microbiol. 74, 345–351. 10.1128/AEM.02076-07 18024678 PMC2223257

[B30] Li M. Malladi S. Hurd H. S. Goldsmith T. J. Brichta-Harhay D. M. Loneragan G. H. (2015). *Salmonella* spp. in lymph nodes of fed and cull cattle: relative assessment of risk to ground beef. Food Control 50, 423–434. 10.1016/j.foodcont.2014.09.011

[B31] McLaughlin J. B. Castrodale L. J. Gardner M. J. Ahmed R. Gessner B. D. (2006). Outbreak of multidrug-resistant *Salmonella* Typhimurium associated with ground beef served at a school potluck. J. Food Protect. 69, 666–670. 10.4315/0362-028X-69.3.666 16541701

[B32] Morley P. S. Dargatz D. A. Hyatt D. R. Dewell G. A. Patterson J. G. Burgess B. A.. (2011). Effects of restricted antimicrobial exposure on antimicrobial resistance in fecal *Escherichia coli* from feedlot cattle. Foodb. Pathog. Dis. 8, 87–98. 10.1089/fpd.2010.0632 21034271

[B33] Nucera D. M. Maddox C. W. Hoien-Dalen P. Weigel R. M. (2006). Comparison of API 20E and invA PCR for identification of *Salmonella enterica* isolates from swine production units. J. Clin. Microbiol. 44, 3388–3390. 10.1128/JCM.00972-06 16954281 PMC1594722

[B34] Olafson P. U. Brown T. R. Lohmeyer K. H. Harvey R. B. Nisbet D. J. Loneragan G. H.. (2016). Assessing transmission of *Salmonella* to bovine peripheral lymph nodes upon horn fly feeding. J. Food Protect. 79, 1135–1142. 10.4315/0362-028X.JFP-15-414 27357032

[B35] Rahn K. Degrandis S. A. Clarke R. C. McEwen S. A. Galan J. E. Ginocchio C.. (1992). Amplification of an invA gene sequence of *Salmonella* Typhimurium by polymerase chain reaction as a specific method of detection of *Salmonella*. Mol. Cell. Prob. 6, 271–279. 10.1016/0890-8508(92)90002-F 1528198

[B36] Rao S. Van Donkersgoed J. Bohaychuk V. Besser T. Song X.-M. Wagner B.. (2010). Antimicrobial drug use and antimicrobial resistance in enteric bacteria among cattle from Alberta feedlots. Foodb. Pathog. Dis. 7, 449–457. 10.1089/fpd.2009.0400 19958100

[B37] Rodriguez-Rivera L. D. Cummings K. J. Loneragan G. H. Rankin S. C. Hanson D. L. Leone W. M.. (2016). *Salmonella* prevalence and antimicrobial susceptibility among dairy farm environmental samples collected in Texas. Foodb. Pathog. Dis. 13, 205–211. 10.1089/fpd.2015.2037 26954516

[B38] Scallan E. Hoekstra R. M. Angulo F. J. Tauxe R. V. Widdowson M. A. Roy S. L.. (2011). Foodborne illness acquired in the United States-major pathogens. Emerg. Infect. Dis. 17, 7–15. 10.3201/eid1701.P11101 21192848 PMC3375761

[B39] Schneider J. White P. Weiss J. Norton D. Lidgard J. Gould L.. (2011). Multistate outbreak of multidrug-resistant *Salmonella* Newport infections associated with ground beef, October to December 2007. J. Food Protect. 74, 1315–1319. 10.4315/0362-028X.JFP-11-046 21819658

[B40] Sjölund-Karlsson M. Joyce K. Blickenstaff K. Ball T. Haro J. Medalla F. M.. (2011). Antimicrobial susceptibility to azithromycin among *Salmonella enterica* isolates from the United States. Antimicrob. Agents Chemother. 55, 3985–3989. 10.1128/AAC.00590-11 21690279 PMC3165283

[B41] Sofos J. N. Kochevar S. L. Bellinger G. R. Buege D. R. Hancock D. D. Ingham S. C.. (1999). Sources and extent of microbiological contamination of beef carcasses in seven United States slaughtering plants. J. Food Protect. 62, 140–145. 10.4315/0362-028X-62.2.140 10030632

[B42] Sorensen O. Van Donkersgoed J. McFall M. Manninen K. Gensler G. Ollis G. (2002). *Salmonella* spp. shedding by Alberta beef cattle and the detection of *Salmonella* spp. in ground beef. J. Food Protect. 65, 484–491. 10.4315/0362-028X-65.3.484 11899047

[B43] Suez J. Porwollik S. Dagan A. Marzel A. Schorr Y. I. Desai P. T.. (2013). Virulence gene profiling and pathogenicity characterization of non-typhoidal *Salmonella* accounted for invasive disease in humans. PLoS ONE 8:e58449. 10.1371/journal.pone.0058449 23505508 PMC3591323

[B44] Wells S. Fedorka-Cray P. Dargatz D. Ferris K. Green A. (2001). Fecal shedding of *Salmonella* spp. by dairy cows on farm and at cull cow markets. J. Food Protect. 64, 3–11. 10.4315/0362-028X-64.1.3 11198437

[B45] Wheeler T. L. Kalchayanand N. Bosilevac J. M. (2014). Pre- and post-harvest interventions to reduce pathogen contamination in the U.S. beef industry. Meat Sci. 98, 372–382. 10.1016/j.meatsci.2014.06.026 25027798

