# Supplementary material for: Salmonella in Peripheral Lymph Nodes of Healthy Cattle at Slaughter
Source: Front Microbiol. 2017 Nov 9;8:2214. doi: 10.3389/fmicb.2017.02214 (PMC5684184; doi:10.3389/fmicb.2017.02214)
Supplement: Supplementary file 1 [file Table1.DOCX]

Supplementary Material

*Salmonella* in Peripheral Lymph Nodes of Healthy Cattle at Slaughter

**Hattie E. Webb^1#^, Dayna M. Brichta-Harhay^2#^, Mindy M. Brashears^1^, Kendra K. Nightingale^1^, Terrance M. Arthur^2^, Joseph M. Bosilevac^2^, Norasak Kalchayanand^2^, John W. Schmidt^2^, Rong Wang^2^, Sophie A. Granier^3^, Tyson R. Brown^4^, Thomas S. Edrington^5^, Steven D. Shackelford^2^, Tommy L. Wheeler^2^, Guy H. Loneragan^1^***

*** Correspondence:** Guy H. Loneragan guy.loneragan@ttu.edu

# Supplementary Figures and Tables

**Table S1. Interpretation criteria used to generate phenotypic resistance patterns as defined by the Clinical and Laboratory Standards Institute (CLSI) M100-S23 unless otherwise indicated**

| Antimicrobial | Abbreviation | Susceptible*^a^* | Resistant | Antimicrobial Class |
| --- | --- | --- | --- | --- |
| Gentamicin | GEN | < 16 | ≥ 16 | Aminoglycosides |
| Kanamycin | KAN | < 64 | ≥ 64 | Aminoglycosides |
| Streptomycin*^b^* | STR | < 64 | ≥ 64 | Aminoglycosides |
| Ampicillin | AMP | < 32 | ≥ 32 | β-lactam |
| Augmentin | AUG | < 32 | ≥ 32 | β-lactam/ β-lactamase Inhibitor combinations |
| Cefoxitin | FOX | < 32 | ≥ 32 | β-lactam |
| Ceftiofur*^b^* | TIO | < 8 | ≥ 8 | β-lactam |
| Ceftriaxone | AXO | < 4 | ≥ 4 | β-lactam |
| Azithromycin*^c^* | AZI | < 16 | ≥ 16 | Macrolides |
| Chloramphenicol | CHL | < 32 | ≥ 32 | Phenicols |
| Ciprofloxacin | CIP | < 1 | ≥ 1 | Quinolones |
| Nalidixic acid | NAL | < 32 | ≥ 32 | Quinolones |
| Sulfisoxazole*^b^* | FIS | < 256 | ≥ 256 | Folate Pathway Inhibitors |
| Trimethoprim-Sulfamethoxazole | SXT | < 4 | ≥ 4 | Folate Pathway Inhibitors |
| Tetracycline | TET | < 16 | ≥ 16 | Tetracyclines |

*^a^*includes susceptible and intermediate; *^b^*criteria from The National Antimicrobial Resistance Monitoring System, Manual of Laboratory Methods, 2011; *^c^*criteria recommended by Sjölund-Karlsson et al. (2011)
